# Gene therapy strategies in ophthalmology—an overview of current developments and future prospects

**DOI:** 10.1007/s13353-025-00973-5

**Published:** 2025-06-05

**Authors:** Julia B. Krajewska, Arleta Waszczykowska

**Affiliations:** 1 Department of Ophthalmology, Norbert Barlicki Memorial Teaching Hospital No. 1, Lodz, Poland; 2 Department of Ophthalmology, Medical University of Lodz, Kopcinskiego 22, 90-153 Lodz, Poland

**Keywords:** Gene therapy, Gene editing, Ocular diseases, Drug development

## Abstract

Gene therapies have recently emerged as promising strategies for treatment of previously incurable or poorly managed diseases. These hopes are particularly high in ophthalmology, as the eye is considered exceptionally suited for gene therapy. Expansion of gene therapy products may change the clinical course of treatment and give new chances to many patients. In this review, we address treatment possibilities and delivery methods as well as challenges and uncertainties related to gene therapy. We present inherited and acquired diseases which are subject to studies within this area, summarize current trends in ocular gene therapy, and indicate the future directions.

## Introduction

Although the first gene therapy products were registered in the twenty-first century, the concept of using gene transfer to treat diseases was introduced in the 1960 s. The first exogenous DNA transfer into mammalian cells, performed by Elizabeth and Waclaw Szybalski in 1962, is often regarded as the starting point of this field (Szybalska and Szybalski 1962; Cring and Sheffield 2020). The concept has been evolving throughout the years and led to the development of gene therapy products that are clearly defined both by the European Medicines Agency (EMA) and the Food and Drug Administration (FDA). According to EMA, gene therapy medicinal product (GTMP) is a “biological medicinal product that contains an active substance which contains or consists of a recombinant nucleic acid used in or administered to humans to regulate, repair, replace, add or delete genetic sequences and its therapeutic, prophylactic or diagnostic effect relates directly to the recombinant nucleic acid sequence it contains, or to the product of genetic expression of this sequence,” while FDA defines gene therapy as products that “mediate their effects by transcription and/or translation of transferred genetic material and/or by integrating into the host genome and that are administered as nucleic acids, viruses, or genetically engineered microorganisms” (Hanna et al. 2017; Goswami et al. 2019). These definitions point to the variety of possibilities given by the evolving field of gene therapies.

Out of the few fully developed gene therapy products, one medication targets an eye disease. Voretigene neparvovec-rzyl, with the trade name of Luxturna is used in patients with confirmed biallelic RPE65 mutation-associated retinal dystrophy. It was approved by the FDA in 2017 and the EMA in 2018. Furthermore, intensive research and robust development have already led to numerous clinical trials, with many more concepts still in the pre-clinical stage. A wide variety of techniques, vectors, and strategies that can be used give hope for many more products that can be introduced in the coming years.

The idea behind gene therapy is to introduce genetic material into the patient’s cells with the use of vectors. Expression of the sequence brings a beneficial health effect. Therapy can be performed ex vivo (isolating the cells of interest from the patient and reintroducing them after genetic modification) or in vivo (delivering the genetic material directly into patient’s tissues) (Lee et al. 2019; Goswami et al. 2019).

## Vectors and delivery routes

A critical element of gene therapy design is selecting the appropriate vector to safely and efficiently deliver the genetic material, achieving optimal long-term gene expression (Solinís et al. 2015; Sahu et al. 2021). An ideal system should have the capacity to transport even large therapeutic genes, ensuring high transduction efficiency as well as stable and long-lasting expression in the targeted cells (mitotic and postmitotic), while random insertion into host genome and ectopic expression must be avoided. It should not cause an inflammatory response and should be well-tolerated, non-immunogenic, and non-pathogenic. Finally, minimally invasive administration and simple, low-cost, and large-scale production should be possible (Solinís et al. 2015; Goswami et al. 2019). There are two main classes of vectors—viral and non-viral vectors (Solinís et al. 2015; Sahu et al. 2021). Viral vectors are the dominant choice in gene therapy aimed at ophthalmic disorders (Ameri et al. 2023).

Viruses display specificity in infecting cell types (tissue tropism); therefore, vector selection depends on the target cell. Viral vectors used in ocular gene therapy include adenovirus (AV), adeno-associated virus (AAV), retrovirus, and lentivirus (Solinís et al. 2015; Ghoraba et al. 2022).

AV vectors can transfect both dividing and non-dividing cells in various cells and tissues (Solinís et al. 2015; Sahu et al. 2021). While the packaging capacity of ordinary AV vector is around 8 kb, some have been engineered to accommodate much larger inserts, up to 37 kb (Lundstrom 2023). Since AVs do not integrate into host genome but remain episomal, the risk of mutagenesis is avoided, but the transgene expression is relatively short. Unfortunately, AVs are common human pathogens, and they often induce immune response (Solinís et al. 2015; Lundstrom 2023).

AAVs are small, non-pathogenic viruses, popularly used as vectors due to their low immunogenicity and wide tropism (Cring and Sheffield 2020; Sahu et al. 2021). Like AVs, they can transfect both dividing and non-dividing cells and usually remain in an extrachromosomal state (Lundstrom 2023). Unfortunately, AAVs have a small carrying capacity of approximately 5 kb (Cring and Sheffield 2020). The viral capsid plays a critical role in determining the efficiency, specificity, and onset of transgene expression in adeno-associated virus (AAV) vectors. Different AAV serotypes exhibit distinct cellular tropism, transduction efficiency, and immunogenicity due to variations in their capsid structure. To enhance vector performance, capsid proteins from different AAV serotypes can be exchanged, creating recombinant or pseudotyped AAVs (e.g., AAV2/8 and AAV2/9). This approach allows for the development of optimized vectors with improved targeting of specific cell types, enhanced transduction efficiency, and reduced immunogenicity, ultimately advancing the effectiveness of gene therapy applications (Solinís et al. 2015; Rodrigues et al. 2018). AAVs have predominantly been chosen as the vectors in ocular gene therapy (Rodrigues et al. 2018).

Retroviruses are RNA viruses that reverse transcribe their RNA into double-stranded DNA, which can integrate into the host genome. This approach is advantageous for long-term transgene expression but creates a risk of insertional mutagenesis and oncogene activation—even resulting in leukemia development in SCID-X1 patients treated in a clinical trial. They have a packaging capacity of 8 kb, low immunogenicity, and broad tropism. Retroviral vectors can only transduce dividing cells (Sahu et al. 2021; Lundstrom 2023).

Lentiviruses belong to the retrovirus family. They are integrating vectors that can infect both dividing and non-dividing cells and have the capacity of approximately 9 kb (Rodrigues et al. 2018; Sahu et al. 2021). Since integrational mutagenesis remains a concern, modifications in lentiviral vectors have been introduced. For example, self-inactivating vectors and nonintegrating vectors have been presented as safer alternatives (Solinís et al. 2015; Rodrigues et al. 2018).

Although non-viral vectors are currently uncommon, these delivery methods are continuously being developed since they show several advantages over viral vectors. Besides their lower production cost, non-viral vectors have higher genetic cargo packing capacity, which are relatively safer (minimal risk of insertional mutagenesis and fewer side effects) and less immunogenic. Unfortunately, their success is mainly limited by the low gene delivery efficiency and uncertainty about expression stability (Sainz‐Ramos et al. 2021; Sahu et al. 2021; Liu et al. 2023). The non-viral strategies include physical methods (such as microinjection, gene gun, electroporation, sonoporation, or magnetofection) and chemical methods (which include lipid-based nanoparticles (NPs), peptide-based NPs, and polymer-based NPs) (Mehier-Humbert and Guy 2005; Sainz‐Ramos et al. 2021; Sahu et al. 2021). Lipid nanocarriers have already proved greatly successful serving to deliver a formulated mRNA vaccine against the SARS-CoV-2 virus (in the products developed by Pfizer-BioNTech and Moderna). These vaccines are a clear example of difficulties related to new technologies: the low shipping and storage temperatures necessary due to mRNA instability proved to limit availability of the products. Hopefully, further development may overcome these shortcomings—e.g., by obtaining a stable dry powder through freeze drying (Mascellino et al. 2021; Sainz‐Ramos et al. 2021). More details regarding lipid nanoparticle technology for ocular gene therapy can be found in a comprehensive review performed by Wang et al. (2024).

**Fig. 1:**
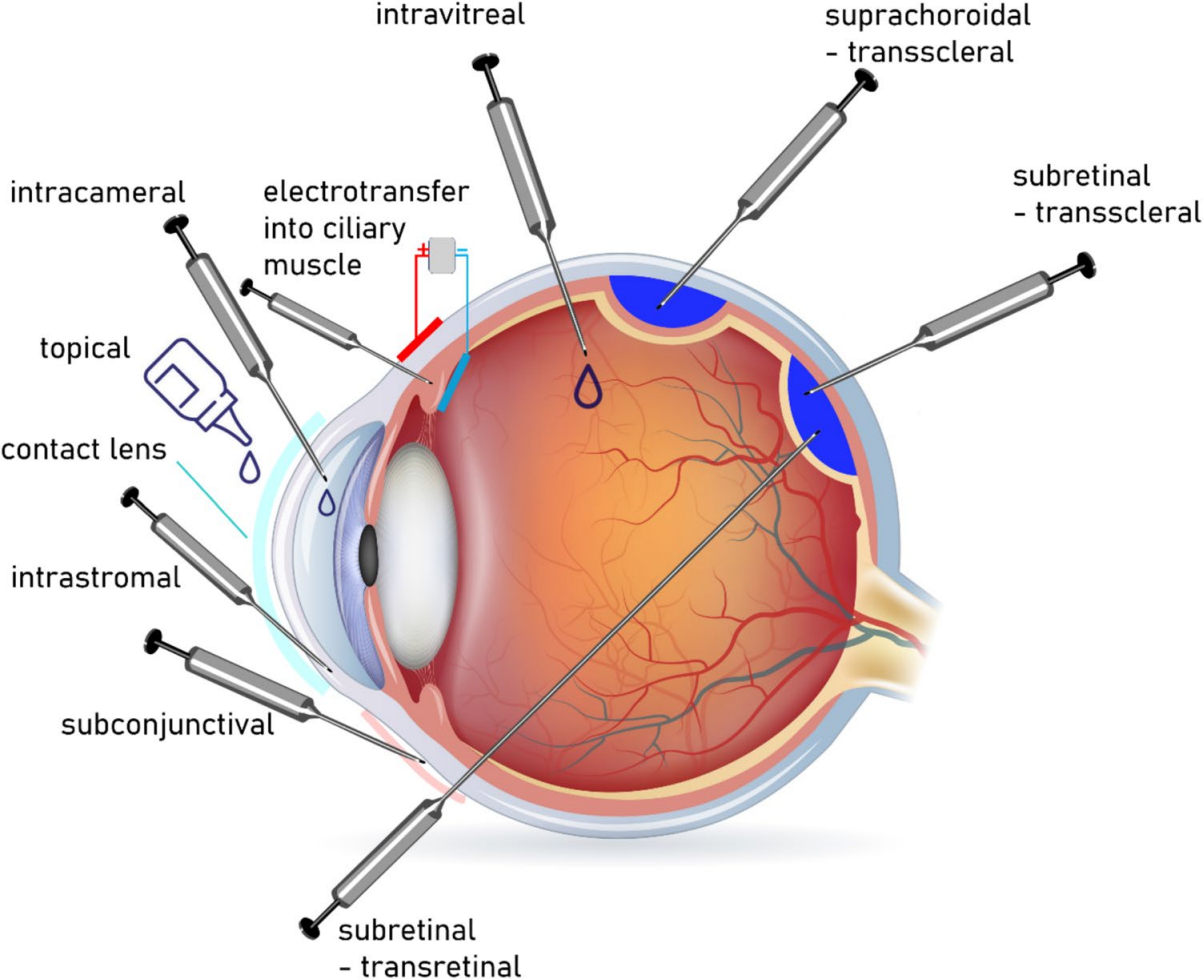
Gene therapy delivery routes in the treatment of ocular diseases

There are several gene therapy administration routes available in the treatment of ocular diseases (Fig. 1). The choice of the appropriate delivery method depends on the eye area and targeted cells as well as the characteristics of the vector (Solinís et al. 2015; He et al. 2023). *Topical administration* is simple and non-invasive, but due to its low intraocular bioavailability, it is primarily used for treating the anterior segment of the eye. While it also serves as a potential route for siRNA gene therapy, challenges such as poor penetration remain (Baran-Rachwalska et al. 2020). Blockade of β-adrenergic receptors in the ciliary epithelium lowers intraocular pressure (IOP) by inhibiting aqueous humor production, which is used in glaucoma treatment. SYL040012, siRNA that specifically inhibits synthesis of β2-adrenergic-receptor, was examined as a potential treatment of elevated IOP. In a study on 25 healthy subjects, topical administration of SYL040012 on a daily basis over a period of 7 days reduced IOP values in 15 out of 24 healthy subjects (Moreno-Montañés et al. 2014). Since naked siRNA molecules face significant barriers to ocular penetration, ProSilic®—a hybrid silicon-lipid nanoparticle system—was developed to enhance delivery. ProSilic® successfully delivered siRNA to the cornea, achieving potent gene silencing. In a murine reporter model with luciferase expression restricted to the corneal epithelium, topical application of ProSilic-DSC613G reduced luciferase expression by 41%, with effects persisting throughout the 8-day treatment regimen and for 4 days post-treatment (Baran-Rachwalska et al. 2020). Viral vectors can also be utilized for topical gene therapy. In a 6-month in vivo study, researchers assessed the safety of topical AAV5-decorin gene transfer in a rabbit model of corneal fibrosis and neovascularization, administering the treatment following a minor corneal epithelial scrape. The findings provide crucial long-term safety data for this promising approach, which harnesses decorin’s natural ability to regulate collagen and inhibit TGF-β, underscoring its potential for treating corneal scarring and vascularization (Mohan et al. 2021). Building upon this research, a subsequent study in 2024 explored the potential of a combination gene therapy using topical AAV5 to deliver both decorin and pigment epithelium-derived factor (PEDF) in a rabbit model of chemically induced corneal fibrosis and neovascularization, demonstrating significant reductions in both conditions with good tolerability. This later study suggests that a multi-gene approach could offer enhanced therapeutic benefits for complex corneal pathologies (Mohan et al. 2024).

Another study presented an approach which holds promise for improving the efficacy and compliance of ocular gene therapy for various corneal diseases. Functionalized hydrogel *contact lenses* were developed as a novel platform for the controlled delivery of rAAV vectors to the cornea, representing a patient-friendly alternative to traditional methods like eye drops and injections. By modifying the contact lens material, the researchers significantly enhanced viral vector loading and enabled sustained release, leading to effective gene transduction in both cell cultures and bovine cornea explants (Alvarez-Rivera et al. 2020).

*Intrastromal injection*, a method where gene therapy agents are directly injected into the corneal stroma, allows for efficient transduction of corneal tissues. Importantly, this technique can enable retrograde transport of the therapeutic agent to the trigeminal ganglion (TG), which is crucial for treating conditions like herpes (Yin et al. 2021; Wei et al. 2023; Cong et al. 2024) In neurotrophic keratopathy (NK), a single intrastromal injection of AAV carrying the nerve growth factor (NGF) gene showed comparable therapeutic effects to frequent recombinant human NGF (rhNGF) eye drops, suggesting it could be a more convenient and long-lasting treatment (Cong et al. 2024). For herpes simplex keratitis (HSK), intrastromal injection of HSV-1-erasing lentiviral particles (HELP), a CRISPR-based therapy, demonstrated the ability to reduce viral load in the cornea and potentially target the latent reservoir in the trigeminal ganglia. Early clinical trials of HELP delivered via intrastromal injection in patients with severe HSK showed acceptable safety with no detectable off-target effects or systemic adverse events in most cases (Yin et al. 2021; Wei et al. 2023).

Touchard et al. proposed an unconventional non-viral gene therapy approach using *electrotransfer* to deliver plasmids encoding therapeutic proteins into the ciliary muscle. In this method, plasmids are injected into the ciliary muscle, followed by short electrical pulses that enhance cellular uptake, transforming the muscle cells into a sustained, localized biofactory for therapeutic protein production within the eye (Touchard et al. 2018).

While *subconjunctival delivery* shares some limitations with topical administration, it can be effective in certain applications. Expanding the use of gene therapy for corneal conditions, Gilger et al. (2024) investigated an alternative approach, demonstrating that subconjunctival injection of AAV8 carrying a single-chain immunomodulator (scIM) significantly inhibited corneal neovascularization in a murine corneal burn model. This study suggests that alternative delivery methods can effectively target key pathways involved in corneal pathology, such as vascularization and fibrosis. The scIM protein was designed to mimic the immunomodulatory effects of HLA-G, an immune checkpoint molecule that promotes immune privilege and inhibits angiogenesis (Gilger et al. 2024).

*Injection into the anterior chamber* is an uncommon choice, though may be used, e.g., when targeting trabecular meshwork or corneal endothelial cells. Rao et al. (2025) investigated intracameral injection of AAV-IKV, a novel adeno-associated virus vector, as a gene therapy delivery route for glaucoma, with relevance to corneal tissues due to its efficient transduction of the anterior segment. Their study demonstrated that intracameral AAV-IKV successfully delivered genes to the ciliary body, corneal stroma, trabecular meshwork, and even some corneal nerves. To model glaucoma pathology, intracameral AAV-IKV-mediated delivery of TGFβ2 CS, a constitutively active form of TGFβ2, elevated IOP and induced trabecular meshwork fibrosis. However, AAV-IKV-mediated delivery of decorin, a TGFβ2 inhibitor, reversed these effects, lowering IOP, reducing fibrosis, and preventing retinal ganglion cell degeneration (Rao et al. 2025).

*Intravitreal, subretinal, and suprachoroidal injections* are most commonly used in order to access the posterior segment, among which the subretinal route has been exploited most widely in clinical trials. These invasive methods, while most effective, have significant limitations (such as the cost, availability of experienced staff, and appropriate equipment) and carry the risk of serious adverse effects (Solinís et al. 2015; He et al. 2023). Suprachoroidal delivery involves injecting therapeutic agents into the potential space between the choroid and sclera. This method results in diffuse, peripheral transgene expression mainly in RPE cells. Intravitreal delivery introduces viral vectors directly into the vitreous cavity via needle injection through the pars plana. This approach often shows minimal transduction of outer retinal cells due to the internal limiting membrane barrier, with expression limited to inner layers or the peripapillary region. Subretinal delivery aims to place viral vectors between the photoreceptors and the RPE. Transretinal subretinal injection requires invasive vitrectomy surgery. It leads to focal gene expression with transduction of photoreceptors and RPE and therefore has a limited therapeutic region. Transscleral subretinal injection uses transscleral access the subretinal space without the need for vitrectomy. Similar to the transretinal approach, it produces focal gene expression in RPE and photoreceptors. Transscleral subretinal delivery is less invasive and easier to perform than transretinal subretinal injection (Yiu et al. 2020). Yiu et al. (2020) investigated ocular gene delivery in rhesus macaques using transscleral microneedles to inject AAV8 into the suprachoroidal or subretinal space, demonstrating the feasibility of this approach and the distinct expression patterns associated with each technique.

Although *systemic administration* could be utilized, local delivery is usually the preferred option. Since the blood-retinal barrier limits drug penetration into ocular tissues and large doses increase the risk of potential off-target effects, systemic administration is avoided (Solinís et al. 2015; He et al. 2023; Appell et al. 2024).

## Genome editing technologies

In the majority of studies, the therapeutic effect is achieved by supplementing genes to produce functional proteins (gene addition) or through inhibiting the expression of the defective gene (gene inhibition–RNA interference therapy). In some diseases, these solutions are insufficient or unsatisfactory. New opportunities have emerged with the introduction of genome editing tools, which have made in vivo gene repair possible (Tang and Xu 2020; Sainz‐Ramos et al. 2021; He et al. 2023; Choi et al. 2023). The discovery that CRISPR-Cas9 system, derived from the bacterial adaptive immune system, can be used as a genetic engineering tool in mammalian cells was revolutionary (Doudna and Charpentier 2014). It is now the most popular genome editing technology due to its simplicity, efficiency, and versatility. CRISPR-Cas9 system consists of two components: a single-guide RNA which recognizes a specific DNA sequence and the Cas9 protein—an endonuclease that can cut the DNA introducing a site-specific double-strand break, which is subsequently repaired by the endogenous repair machinery, introducing modifications at the cut site (Doudna and Charpentier 2014; Choi et al. 2023).

The first in vivo CRISPR-based therapy was administered to an adult patient with Leber congenital amaurosis type 10, a condition caused by a point mutation in the *CEP290* gene (First CRISPR therapy dosed 2020). EDIT-101 is a gene-editing therapy that uses an AAV5 vector to deliver *Staphylococcus aureus* Cas9 and CEP290-specific guide RNAs to photoreceptor cells via subretinal injection, aiming to restore normal *CEP290* expression for the treatment of Leber congenital amaurosis type 10 (Maeder et al. 2019). In the phase I/II clinical trial, EDIT-101 was administered to 14 patients via a subretinal injection in the worse (study) eye. According to the Editas Medicine announcement from November 2022, EDIT-101 showed no serious adverse events or dose-limiting toxicities; however, only three patients achieved clinically marked visual improvement (He et al. 2023). In an article presenting the trial results published in May 2024, the authors reported that six participants had a meaningful improvement from baseline in the vision-related quality-of-life score. These outcomes demonstrate that CRISPR-based gene editing therapeutic can be delivered safely and bring clinical benefit, which supports further attempts to develop gene editing–based treatment methods (Pierce et al. 2024).

A gene-editing strategy was also employed in a clinical trial evaluating the safety and efficacy of HELP, a therapeutic product that specifically cleaves two essential genes critical for the HSV-1 life cycle, thereby eliminating the virus. Lentiviral particles targeting HSV-1 by CRISPR were delivered through intrastromal injection during penetrating keratoplasty in three patients with severe refractory herpetic stromal keratitis and acute corneal perforation. Corneal grafts and tear swabs were free of viral relapse in the 18 months follow-up (despite discontinuation of the antiviral therapy after HELP administration), and no off-target cleavages in the human genome or adverse events were described (Yin et al. 2021; Wei et al. 2023).

## Eye as the organ predisposed for gene therapy

The eye is often recognized as an excellent target for gene therapy. There are several arguments for its superiority over other organs. The eye’s small size and enclosed space allow for achieving a therapeutic effect through the local administration of a minor amount of the drug. The blood-retinal barrier generates a relatively immune-privileged environment, limiting the systemic exposure of the drug, immune responses, and inflammatory reactions. The eye is easily accessible which enables accurate delivery as well as direct and straightforward examination. Moreover, in a single patient, one eye can serve as the experimental target, while the other can act as a control. Finally, since individual or multiple genes responsible for many ocular disorders have been identified, ophthalmology provides suitable target diseases. Targeting the post-mitotic retinal cells allows for sustained gene expression without genomic integration of the transgene. Additionally, gene therapy offers the key advantage of long-lasting treatment. While it also requires invasive delivery, it has the potential to surpass conventional therapies like anti-vascular endothelial growth factor (VEGF) injections by providing a sustained therapeutic effect, reducing the need for frequent interventions and minimizing the risks and adverse events associated with repeated intravitreal injections (Rodrigues et al. 2018; Lee et al. 2019; Ghoraba et al. 2022; Wasnik and Thool 2022).

The immune privilege of the eye arises from multiple factors that suppress inflammation and promote immune tolerance. Physical barriers, such as the blood-ocular barrier formed by tight junctions of endothelial and RPE cells, restrict immune cell migration, while the lack of lymphatic drainage further limits immune responses. Additionally, the eye produces immunosuppressive molecular regulators that promote tolerance by modulating antigen-presenting cells (APCs), regulatory T cells, and microglia.

This immune privilege is strong enough to support indefinite survival of histoincompatible grafts in the anterior chamber, which led to its initial characterization. It also facilitates long-term corneal transplant success by inducing antigen-specific tolerance rather than inflammatory responses. Similarly, the ocular microenvironment enables sustained survival of transplanted embryonic stem cells in the subretinal space. These features are particularly advantageous for gene therapy, as they limit immune responses against viral vectors, potentially allowing for repeated treatments without significant immune rejection (Taylor and Ng 2018).

While the eye has immune privilege, it is not absolute, and AAV vector delivery can still elicit immune responses. Yiu et al. (2020) analyzed the immunological impact of AAV gene therapy across different ocular compartments, finding that suprachoroidal AAV8 induced localized inflammation in the outer retina and choroid but triggered a lower systemic antibody response. In contrast, subretinal AAV8, delivered transsclerally, caused minimal local inflammation, and did not significantly contribute to systemic antibody production compared to suprachoroidal delivery in the same animals. Although previous studies suggested that intravitreal delivery induces more intraocular inflammation than subretinal delivery, Yiu et al. found that intravitreal AAV8 elicited a stronger systemic humoral response while causing less local inflammation (Yiu et al. 2020). Maidana et al. (2025) found that transscleral injection induces a more pronounced early infiltration of CD11b +, CCR2 +, Ly6G + leukocytes, and macrophage/microglia-like cells, while transvitreal delivery shows higher Ly6 C + monocyte infiltration. Furthermore, the study revealed that the transscleral approach leads to higher photoreceptor cell death in the late stages compared to the transvitreal method, despite both inducing similar levels of reactive gliosis (Maidana et al. 2025). Beyond differences in administration routes, vector immunogenicity is also dose-dependent, with innate immunity playing a key role in the outcome of gene transfer (Verdera et al. 2020).

Clare et al. (2025) analyzed how age and sex influence the ocular immune response to AAV. The early response to AAV involves activation of resident microglia, antigen presentation, and lymphocyte infiltration. While clinical inflammation may resolve, subclinical inflammation with elevated intraocular immune cells can persist. The study found that older age is associated with increased and persistent inflammation. Females, particularly older females, can exhibit a more pronounced and dysregulated microglial response that may contribute to retinal degeneration. The findings have implications for relative AAV safety depending on sex and age, particularly given that women, who are disproportionately affected by age-related macular degeneration (AMD) and glaucoma, may respond differently to gene therapy (Clare et al. 2025).

Gene therapy–associated uveitis (GTAU) refers to intraocular inflammation triggered by innate and adaptive immune responses against vector components following ocular gene therapy. This inflammatory reaction poses a significant clinical concern, as it can lead to retinal cell loss, visual function deterioration, and reduced treatment durability, particularly in one-time gene therapy applications, where long-term efficacy is crucial. Further research into the underlying mechanisms of GTAU may enable the development of targeted immunomodulatory strategies to mitigate its risk and severity, ultimately improving the safety and clinical outcomes of ocular gene therapy (Purdy et al. 2025).

## Ocular gene therapy research and trials—growing interest

By the end of 2022, there were 159 gene therapy clinical trials targeting ophthalmic disorders. The first one, targeting gyrate atrophy, started in 1998. The great majority (96%) was related to retina and optic nerve disorders. USA proved to be dominant in this area: 71% of the trials were conducted in the USA (either exclusively or as one of the countries participating in multinational trials) (Ameri et al. 2023). As of early March 2025, a search on ClinicalTrials.gov, the most comprehensive clinical trial registry, identified 351 studies on gene therapy for ophthalmic diseases. Among them, 20 had not yet begun recruitment, 160 were ongoing, 118 were completed, and 22 had been withdrawn or terminated (ClinicalTrials.gov). So far, the research resulted in one currently available FDA-approved product only, which illustrates the difficulties of gene therapy drug development (Ameri et al. 2023).

The first gene therapy product approved for medical use among humans was fomivirsen, known commercially as Vitravene. However, it is not always classified as a gene therapy in the traditional sense, as it does not modify or replace genes but instead functions as an antisense oligonucleotide that blocks viral mRNA. It received FDA approval for the treatment of cytomegalovirus (CMV) retinitis in AIDS patients on 26 August 1998. Fomivirsen was designed to inhibit viral protein synthesis through binding to CMV mRNA and subsequently prevent viral replication. The drug was administered through intravitreal injections and though it showed effectiveness, it was discontinued in Europe in 2002 and USA in 2006 due to the development of highly effective antiretroviral therapies and a dramatic decrease in CMV retinitis cases among AIDS patients (Yu and Tu 2022; Alhamadani et al. 2022).

**Fig. 2:**
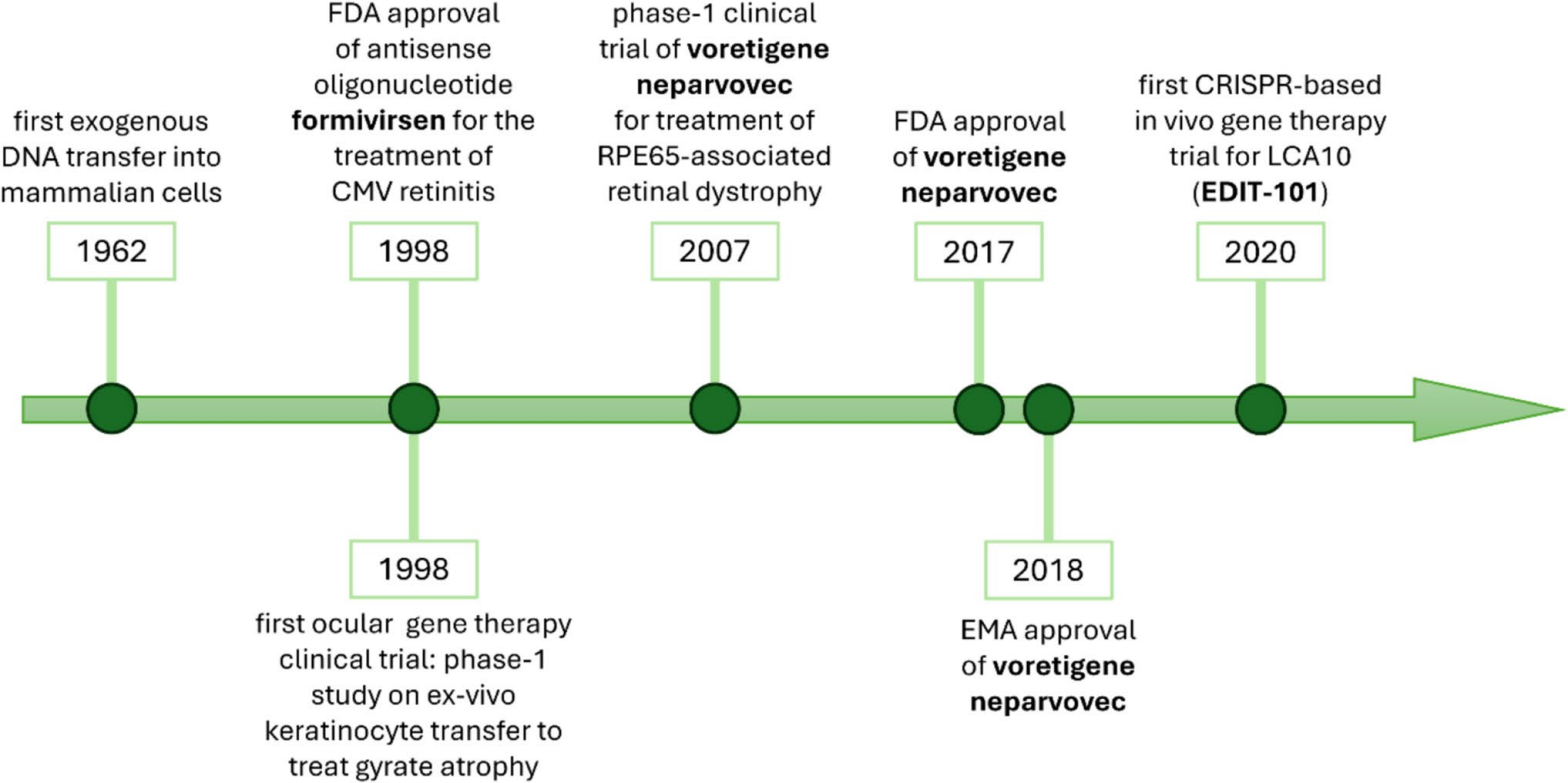
Timeline of milestones in the development of ocular gene therapy

Another milestone was marked with the approval of the first ocular gene therapy, voretigene neparvovec (VN), for the treatment of RPE65-associated retinal dystrophy, first by the FDA in 2017 and then EMA in 2018 (Testa et al. 2024) (Fig. 2). RPE65 is an enzyme expressed in the retinal pigment epithelium (RPE) cells necessary to maintain the visual cycle. Mutations in both copies of RPE65 disrupt the regular retinoid cycle and block the signal transduction cascade, hindering vision generation. Additionally, accumulation of toxic precursors results in progressive photoreceptor dysfunction and retinal degeneration. VN is a recombinant AAV vector serotype 2 containing human RPE65 cDNA. Injection of VN into the subretinal space induces the production of a functional RPE65 enzyme, allowing for the restoration of the visual cycle (Luxturna EMA Summary of Product Characteristics; Chiu et al. 2021; Sengillo et al. 2022). Spark Therapeutics started the phase 1 clinical trial of VN in 2007, and since the results were favorable, the evaluation progressed to subsequent phases. Finally, based on the positive evaluation of safety and efficacy (visual and retinal function improvements), VN was concluded to show a favorable benefit-to-risk profile and entered the market under the commercial name of Luxturna (Maguire et al. 2009, 2019; Bennett et al. 2016; Russell et al. 2017). Since the commercialization, real-world data regarding VN treatment results have been growing, offering new insights into long-term safety and effectiveness. Although the patient population is still very limited, real-world studies provide additional information especially concerning rare adverse events (Sengillo et al. 2022; Deng et al. 2022; Testa et al. 2022; Kiraly et al. 2023; Fischer et al. 2024; Melillo et al. 2024).

Despite initial enthusiasm and numerous positive treatment reports, uncertainties and doubts have emerged over time as the number of treated patients has increased. Firstly, there is currently no established consensus on the eligibility criteria for VN treatment. Specifically, while a sufficient number of viable retinal cells is required for treatment, standardized guidelines for assessing cell viability have not been defined. In their analysis, Testa et al. (2024) concluded that functional rescue depends more on the pre-treatment preservation of photoreceptors than on patient age, but clear indicators for treatment eligibility were not identified (Testa et al. 2024). Secondly, chorioretinal atrophy growth after VN has been reported by several authors in real-life studies, which was not described in clinical trials (Russell et al. 2017; Kiraly et al. 2023; Fischer et al. 2024; Stingl et al. 2024; Ku et al. 2024; Melillo et al. 2024). Atrophy was not associated with worse visual outcomes, but had positive correlation with successful treatment; nevertheless, it is a significant adverse effect whose long-term consequences may be unfavorable (Stingl et al. 2024; Ku et al. 2024; Melillo et al. 2024). Although several explanations are possible, Stingl et al. suggested that the atrophy growth could be a metabolic stress response in the retina due to a change in rod activity (Stingl et al. 2024; Ku et al. 2024). Next, the use of non-routine, custom-designed testing method to assess efficacy can be considered controversial, while high therapy cost may become a financial burden to societies (Darrow 2019). Finally, the treatment outcomes are not homogeneously reported across the studies, which make data analysis and drawing conclusions difficult (Testa et al. 2024).

In addition to uncertainties regarding eligibility and long-term efficacy, voretigene neparvovec has been associated with various adverse events, many of which stem from the surgical procedure rather than the gene therapy itself. Voretigene neparvovec has been associated with various adverse events, many of which stem from the surgical procedure rather than the gene therapy itself. Common but generally transient side effects include elevated intraocular pressure, cataract formation, ocular inflammation, retinal tears, subretinal deposits, pigment displacement, and temporary disorganization of the retina at the injection site (Maguire et al. 2019; Gange et al. 2022). More serious complications have also been reported, including irreversible vision loss in two cases, retinal detachment, subretinal hemorrhage, and subretinal neovascularization, as well as paracentral scotomas related to retinal atrophy in some patients (Maguire et al. 2019; Lorenz 2025).

A particularly concerning post-marketing finding has been progressive perifoveal chorioretinal atrophy (CRA), observed in up to 50% of treated eyes (Lorenz 2025). The exact cause of CRA remains unclear, but potential contributing factors include immune responses, surgical trauma, metabolic imbalances, or increased rod function following treatment (Gange et al. 2022; Stingl et al. 2024; Lorenz 2025). Additionally, an immune response to the AAV vector has been suggested in at least one case, where foveal photoreceptor loss occurred in the second treated eye (Jalil et al. 2022). While Luxturna was initially considered to have a safety profile comparable to standard vitrectomy, the emergence of CRA as a significant long-term concern underscores the need for continued monitoring and research. Despite these risks, many patients experience substantial improvements in visual function, particularly in low-light conditions, emphasizing the importance of balancing the benefits and potential complications of this one-time gene therapy treatment (Lorenz 2025).

Every new pathway inevitably encounters difficulties. However, the challenges and uncertainties that have appeared should not be discouraging. The large number and variety of clinical trials demonstrates potential for new products, which may be introduced in the coming years (Wang et al. 2024). Arabi et al. listed gene therapy products currently waiting for approval from EMA and/or FDA, which include therapies targeting ocular diseases, such as X-linked retinitis pigmentosa, achromatopsia, and choroideremia (Arabi et al. 2022).

## Ocular gene therapy prospects—selected examples

### Inherited retinal diseases

Inherited retinal diseases (IRDs) naturally emerge as prime candidates for gene therapy. So far, there have been no effective treatment options for these blinding disorders, which significantly reduce patients’ quality of life and constitute a major socioeconomic burden (Ng et al. 2024). Additionally, many of these conditions are caused by mutations in a single gene, and these genetic defects have been identified. The research in this area, which is very extensive and exceeds the scope of this article, has been summarized in comprehensive reviews (Hu et al. 2021; Georgiou et al. 2021; Chiu et al. 2021; Brar et al. 2024). While the majority of ocular gene therapy research focuses on IRDs, the potential for treating other eye diseases is equally promising and exciting.

### Glaucoma

Glaucoma is the main cause of blindness in industrialized countries due to progressive optic neuropathy, commonly associated with elevated IOP. Unfortunately, current treatments that focus on lowering IOP are often unsuccessful or insufficient. Gene therapy offers several innovative approaches. Firstly, reducing IOP by decreasing aqueous humor production or enhancing outflow via genetic modulation may prove more effective than using currently available pharmaceuticals. Secondly, gene therapy strategies that provide neuroprotection for retinal ganglion cells (RGCs) could be particularly beneficial for patients who experience disease progression despite controlled IOP (Moreno-Montañés et al. 2014; Sulak et al. 2024).

Three gene therapy products targeting glaucoma have reached clinical trials. The first, bamosiran (SYL040012), is an siRNA molecule that inhibits the synthesis of the β2-adrenergic receptor when administered topically, leading to a significant reduction in IOP. Bamosiran (SYL040012) shows a good tolerability and safety profile (Moreno-Montañés et al. 2014; Gonzalez et al. 2016). The second, QPI-1007, delivered through a single intravitreal injection, is an siRNA that inhibits the pro-apoptotic protein caspase-2, therefore preventing RGC death. The results of this clinical trial have not been reported (Jiang et al. 2021).

In June 2024, a clinical trial began evaluating CRISPR/Cas9 instantaneous gene editing therapy in patients with primary open-angle glaucoma (POAG) who have elevated IOP and a myocilin (*MYOC*) gene mutation (ClinicalTrials.gov: NCT06465537). *MYOC* gene mutations, associated with approximately 4% of adult-onset POAG cases and over 10% of juvenile-onset cases, cause toxic myocilin accumulation in trabecular meshwork cells, impairing aqueous humor outflow and raising IOP (Kwon et al. 2009).

Previous studies in a POAG mouse model using an adenovirus carrying CRISPR (Ad5-crMYOC) administered through intravitreous injection, successfully disrupted the *MYOC* gene and its aberrant function, lowered IOP, and protected against further glaucomatous changes (Jain et al. 2017). The ongoing clinical trial will assess a similar system—CRISPR/Cas9 Instantaneous Gene Editing Therapy (BD113 virus-like particle)—for its safety, tolerability, and preliminary efficacy in POAG patients with MYOC mutations. Participants will receive a single intracameral injection, with follow-up evaluations over 1 year (ClinicalTrials.gov: NCT06465537).

### Age-related macular degeneration

AMD is a leading cause of severe vision loss globally, with its prevalence expected to rise in the coming years. The disease exists in two forms: neovascular AMD (nAMD) and dry AMD. Intravitreal injections of anti-VEGF agents are the standard treatment for nAMD, but despite the significant improvements these therapies offer, they are not without drawbacks.

Frequent clinic visits and repeated intravitreal injections place a substantial burden on patients and healthcare systems, in addition to increasing the risk of adverse events. Additionally, some patients show limited response to treatment (Blasiak et al. 2024; Rowe and Ciulla 2024). Numerous clinical trials are investigating gene therapy for nAMD. While these therapies continue to target VEGF, they aim to improve treatment response and maintain visual gains through prolonged expression, thereby reducing the frequency of treatments and easing patient burden. These options could become available in the near future, thanks to ongoing research and advanced trials (Trincão-Marques et al. 2024; Blasiak et al. 2024; Rowe and Ciulla 2024).

Dry AMD is characterized by RPE dysfunction, photoreceptor loss, and retinal degeneration, with the late stage known as geographic atrophy (GA) (Schultz et al. 2021). Pathways involving the complement system, oxidative stress, and lipid metabolism are implicated in AMD pathogenesis. The first treatments, recently approved in 2023, include pegcetacoplan and avacincaptad pegol, which inhibit the complement cascade via regular intravitreal injections. However, these treatments are limited and insufficient as they have not consistently demonstrated significant improvement in visual acuity, require frequent invasive administrations, and carry potential safety risks and side effects, including the risk of conversion to the neovascular form of the disease (Trincão-Marques et al. 2024; Rowe and Ciulla 2024).

JNJ-1887, delivered intravitreally, uses an AAV2 vector to increase the expression of a soluble form of CD59, an anti-inflammatory protein in the complement pathway, which is under-expressed in retinal cells in the course of GA (Trincão-Marques et al. 2024; Heier et al. 2024; Rowe and Ciulla 2024). OCU410, based on AAV delivery of the *RORA* gene via subretinal injection, addresses both lipid metabolism (reducing lipofuscin deposits and oxidative stress) and the complement system (providing anti-inflammatory effects) (Rowe and Ciulla 2024). GT-005, also delivered subretinally, induces the expression of complement factor I, a natural inhibitor of the complement system, using an AAV2 vector (Trincão-Marques et al. 2024).

### Uveitis

Non-infectious uveitis is primarily treated with corticosteroids, though systemic immunomodulatory therapy, including TNF inhibitors, may be necessary in some cases. A strategy for local administration could achieve therapeutic effects while minimizing the risk of adverse events. A novel gene therapy approach for non-infectious uveitis involves the use of a clinical-grade plasmid DNA, pEYS606, which encodes a fusion protein with high affinity for human TNF-α. This DNA is transduced into ciliary muscle cells via electrotransfer, resulting in the secretion of therapeutic proteins into ocular fluids. The treatment significantly reduced ocular inflammation in two rat models of uveitis, granting advancement to a phase I/II clinical trial (Touchard et al. 2018).

### Corneal diseases

Gene therapy holds promise for treating and potentially curing both inherited and acquired corneal disorders, as well as metabolic diseases affecting the cornea (Klausner et al. 2007). Research has yielded promising results in the prevention and treatment of corneal diseases, as thoroughly reviewed by Amador et al. (2022) and Di Iorio et al. (2019).

The cornea is an attractive target for gene therapy due to its accessibility for agent delivery, the ability to conduct noninvasive examinations, and its relative immune privilege (Amador et al. 2022; Mohan et al. 2024). Various gene delivery strategies, including viral and non-viral vectors, have been explored to address corneal diseases.

Viral vectors, such as AAVs and adenoviruses, are widely used for their high gene transfer efficiency. For example, AAV5 has shown promise for delivering genes into the corneal stroma, while lentiviral vectors are also under investigation (Amador et al. 2022; Mohan et al. 2024). In contrast, non-viral vectors, such as nanoparticles and naked plasmid DNA, offer advantages like lower immunogenicity and ease of manufacturing (Amador et al. 2022).

Advances in gene editing have further expanded therapeutic possibilities. CRISPR-Cas9 is being investigated for genetic corneal dystrophies, enabling precise gene deletion, replacement, or correction. Studies have demonstrated the feasibility of CRISPR/Cas9-mediated editing to repair disease-causing mutations in corneal cells (Taketani et al. 2017). Additionally, antisense oligonucleotides and siRNAs are being explored for targeting specific mutations in dystrophies like Fuchs endothelial corneal dystrophy (Amador et al. 2022).

Beyond genetic disorders, gene therapy is also being developed to modulate corneal fibrosis, wound healing, immune response, and neovascularization. Targeting TGF-β signaling with Smad7, bone morphogenic protein-7, and decorin has shown promise in reducing corneal scarring in animal models (Gupta et al. 2017; Mohan et al. 2024). In rabbits, combination gene therapy with AAV5-DCN and AAV5-PEDF significantly reduced corneal fibrosis (Mohan et al. 2024). Moreover, gene therapy involving c-Met and hepatocyte growth factor has been explored to enhance corneal epithelial wound healing, particularly in diabetic corneas (Amador et al. 2022).

Immunomodulatory gene therapy is also an emerging strategy for prolonging corneal allograft survival. Gene transfer of interleukin-10 and CTLA4-Ig has been investigated for this purpose (Amador et al. 2022). A notable study by Gilger et al. (2024) demonstrated that delivering a chimeric anti-vascularization immunomodulator via AAV8 ex vivo to corneal grafts effectively reduced inflammation and neovascularization in a high-risk corneal transplant model.

Anti-angiogenic gene therapy has also shown potential in inhibiting corneal neovascularization. Genes such as decorin, PEDF, angiostatin, and vasohibin-1, delivered through various vectors, have demonstrated efficacy in suppressing abnormal blood vessel formation (Amador et al. 2022; Mohan et al. 2024). In a chemically injured rabbit cornea model, simultaneous delivery of decorin and PEDF genes via AAV5 significantly reduced both corneal fibrosis and neovascularization while being well tolerated by the ocular tissue (Mohan et al. 2024).

Emerging research is also exploring gene therapy for NK and HSK. In vivo CRISPR gene editing using HELP offers a promising strategy for severe refractory HSK, as it directly targets and reduces the HSV-1 virus in the cornea with an acceptable safety profile (Wei et al. 2023). In another study, Cong et al. (2024) investigated a single intrastromal injection of AAV carrying the NGF gene in various mouse models of NK, including those induced by capsaicin, HSK, type II diabetes, and alkali burns. The researchers found that this approach efficiently transduced corneal nerve fibers and was transported back to the trigeminal ganglion, leading to enhanced corneal nerve repair, accelerated epithelial healing, reduced corneal swelling, and improved corneal sensitivity across all tested NK models (Cong et al. 2024).

## Challenges, difficulties, and future prospects

While gene therapy offers new treatment opportunities and significant clinical benefits, its novel nature brings challenges and uncertainties. Firstly, safety is a primary concern. The severity of immune and inflammatory reactions depends on the choice and dose of vector, as well as the route of administration (Bucher et al. 2021; Ghoraba et al. 2022; Ford et al. 2024). One of the major hurdles in ocular gene therapy is the immune response to the viral vectors or the delivered transgene product, which can manifest as GTAU. This inflammation can lead to irreversible loss of retinal cells, deterioration of visual function, and reduced durability of treatment effect (Tummala et al. 2021; Purdy et al. 2025). Such events may lead to treatment failure, and in rare cases, severe intraocular inflammation can result in permanent vision loss (Bucher et al. 2021; Ghoraba et al. 2022; Ford et al. 2024). Additionally, CRISPR-Cas9-mediated genome editing poses a risk of off-target mutations, potentially leading to oncogenic transformations or functional impairments (Cring and Sheffield 2020; Tang and Xu 2020). Safety concerns also include the potential for insertional oncogenesis and the genotoxicity associated with long-term transgene and viral vector expression (Lee et al. 2019; Ghoraba et al. 2022).

Another challenge is the genetic heterogeneity of diseases targeted by gene therapies. For example, there are at least 25 genes that can independently cause autosomal recessive retinitis pigmentosa, while inherited retinal dystrophies involve mutations in 220 different genes. Identifying the causative gene and developing a specific, targeted product is a costly and complex process (Cring and Sheffield 2020). For inherited retinal diseases, accurate mutation identification and molecular diagnosis are essential prerequisites for effective gene therapy (Lee et al. 2019).

Effective and safe delivery of therapeutic genes to target cells within the eye is a significant challenge. Developing vectors with tissue-selective targeting is a key focus of ongoing research to minimize off-target effects (Solinís et al. 2015). The durability of gene therapy treatment is another uncertainty. Once a therapeutic effect is achieved, the duration of its impact and the persistence of transgene expression remain unclear. Achieving long-term gene expression is essential for sustained treatment benefits, and the durability of the therapeutic effect is critical to successful outcomes (Solinís et al. 2015; Lee et al. 2019). Current data from animal studies and early human trials suggest that expression can be maintained for over 10 years, but more clinical information will become available over time (Muhuri et al. 2022). Strategies which allow for repeat dosing of gene therapy are being explored to address cases where the therapeutic effect diminishes or when new genetic targets emerge (e.g., when cancers evolve over time). Finally, the high cost of gene therapy can also pose a significant barrier to patient access, limiting the availability of these treatments (Lee et al. 2019).

Despite various challenges, the rapidly advancing field of gene therapy presents promising future prospects that could offer transformative benefits to patients. Ocular gene therapy is evolving rapidly with the potential to revolutionize treatments for a wide array of eye diseases. The initial successes in treating inherited retinal disorders like Leber congenital amaurosis have paved the way for expanding gene therapy applications to more prevalent conditions (Ghoraba et al. 2022; Ameri et al. 2023). Researchers are increasingly focused on developing novel gene therapy treatments for non-hereditary ocular diseases such as nAMD, glaucoma, and diabetic retinopathy. By providing sustained therapeutic effects, gene therapy could significantly reduce the need for frequent interventions, such as anti-VEGF injections for AMD (Rodrigues et al. 2018; Wasnik and Thool 2022; Rowe and Ciulla 2024).

Furthermore, advancements in genome editing technologies, particularly CRISPR/Cas systems, offer a promising frontier for ocular gene therapy by enabling precise modification or correction of disease-causing genes. Emerging techniques like prime editing and base editing could further enhance the precision of gene modifications (Lee et al. 2019; Trincão-Marques et al. 2024). Continued research is dedicated to developing novel viral vectors with improved tropism for specific retinal cell types and reduced immunogenicity, potentially allowing for less invasive delivery routes, such as intravitreal or suprachoroidal injections. Non-viral delivery methods are also being actively explored to enhance treatment safety and efficiency (Rodrigues et al. 2018; Ghoraba et al. 2022; Wasnik and Thool 2022).

Looking ahead, future strategies aim to develop more targeted immunomodulatory approaches to prevent and treat GTAU, based on a deeper understanding of the underlying immune mechanisms. As research progresses, innovations in ocular gene therapy are expected to result in improved efficacy and safety profiles, expanding the potential application of these therapies to a broader spectrum of ocular diseases (Purdy et al. 2025).

## Abbreviations

AAV: Adeno-associated virus
AV: Adenovirus
AMD: Age-related macular degeneration
CMV: Cytomegalovirus
CRA: Chorioretinal atrophy
EMA: European Medicines Agency
FDA: Food and Drug Administration
GA: Geographic atrophy
GTAU: Gene therapy-associated uveitis
GTMP: Gene therapy medicinal product
HELP: HSV-1-erasing lentiviral particles
HSK: Herpes simplex keratitis
IOP: Intraocular pressure
IRD: Inherited retinal disease
MYOC: Myocilin
NP: Nanoparticle
NGF: Nerve growth factor
NK: Neurotrophic keratopathy
PEDF: Pigment epithelium-derived factor
POAG: Primary open-angle glaucoma
RGC: Retinal ganglion cell
RPE: Retinal pigment epithelium
VN: Voretigene neparvovec
